# Super-oscillatory focusing of circularly polarized light by ultra-long focal length planar lens based on binary amplitude-phase modulation

**DOI:** 10.1038/srep29068

**Published:** 2016-06-29

**Authors:** Gang Chen, Yuyan Li, Anping Yu, Zhongquan Wen, Luru Dai, Li Chen, Zhihai Zhang, Senlin Jiang, Kun Zhang, Xianyou Wang, Feng Lin

**Affiliations:** 1 Key Laboratory of Optoelectronic Technology and Systems (Chongqing University), Ministry of Education, and Key Disciplines Lab of Novel Micro-nano Devices and System Technology, Chongqing University, 174 Shazheng Street, Shapingba, Chongqing 400044, China; 2 National Center for Nanoscience and Technology, No. 11 Zhong Guan CunBei Yi Tiao, Beijing 100190, China; 3 School of Physics, State Key Laboratory for Mesoscopic Physics, Peking University, Beijing 100871, China

## Abstract

In traditional optics, the focal spot size of a conventional lens is restricted to the diffraction limit 0.5*λ*/NA, where *λ* is the wavelength in vacuum and NA is the numerical aperture of the lens. Recently, various sub-diffraction focusing optical devices have been demonstrated, but they usually have short focal length and high numerical aperture. Moreover, they always suffer the problem of huge sidelobes near the focal spot and small field of view, especially when the focal spot size is less than the super-oscillation criteria 0.38*λ*/NA. To address the problem, here, we reported a far-field sub-diffraction point-focusing lens based on binary phase and amplitude modulation with ultra-long focal length 252.8 μm (399.5λ) and small numerical aperture 0.78, and experimentally demonstrated a super-oscillatory focusing of circularly polarized light with spot size 287 nm (0.454λ), smaller than the diffraction limit 0.64λ and the super-oscillation criterion 0.487λ. What’s more, on the focal plane, in the measured area within the radius of 142λ, the largest sidelobe intensity is less than 26% of the central lobe intensity. Such ultra-long distance super-oscillatory focusing with small sidelobes and large field of view has great potential applications in far-field super-resolution microscopy, ultra-high-density optical storage and nano-fabrication.

Optical lenses are fundamental elements for various optical systems. According to the propagation property of electromagnetic waves in lossless medium^1^, the high spatial frequency components are left as evanescent waves, which vanish within a distance of less than one wavelength. Only the components, with spatial frequency less than *n*/λ, propagate into far-field, *n* and λ being the medium refractive index and wavelength in vacuum. The absence of high spatial frequency components in far-field results in the diffraction limit, which restricts the focal spot size of a conventional optical lens to 0.5*λ*/*NA*, where *λ* is the wavelength and *NA* is the numerical aperture of the lens^2^. Usually, evanescent waves are engineered with the help of surface plasmons to realize super-resolution imaging in the near field region of super lens^3^ and plasmonic cavity lens^4^ etc., with demonstrative applications in nano-lithography even beyond the conventional near field diffraction limit^5^. However, evanescent waves decay within a distance less than one wavelength, and fail to meet the requirement for far-field super-resolution applications. Recently, optical super-oscillation^6^ has generated great interest in developing photonic devices to break the diffraction limit in far-field. According to super-oscillation theory, an arbitrary small spot can be constructed utilizing band-limited function^7^, but large sidelobes unavoidably emerge within the area close to such a spot, leaving a very limited field of view. Optical super-oscillatory focusing has been demonstrated through commercialized spatial light modulator^8^, optical eigenmodes^9^,^10^, nano optical antennas^11^, pupil filters^12^, and binary amplitude modulation mask^13^,^14^,^15^. Up to now, a variety of super-oscillatory lens have been proposed and used for super resolution microscopes^7^,^8^,^14^ and telescopes^16^, showing sub-diffraction resolving power with local optical transfer ability^16^ beyond the cut-off frequency defined in conventional Abbe Fourier imaging principle. Among them, binary amplitude modulation mask is the most commonly used method. Unfortunately, strong sidelobes inevitably emerged around such focal spot. Particularly, when a focal spot smaller than the super-oscillation criterion 0.38λ/NA^17^, large sidelobes seem to be inevitable^18^. This presents a great challenge in the development of sub-diffraction photonics devices and their applications. To avoid large sidelobes, for a given focal spot size, the most efficient way is to decrease the value of 0.38λ/NA by increasing NA. Based on this idea, a high NA planar metalens was proposed for sub-diffraction focusing of azimuthally polarized light. A focal spot size of 0.42λ was realized with a planar lens with high NA value of 0.95, which was designed in a non-super-oscillatory way for the purpose of sidelobe suppression, however, the maximum sidelboe intensity is 40% of the central spot^18^. Recently, a super-oscillatory planar lens, based on quasi-continuous amplitude modulation, has been demonstrated with sub-wavelength metallic slit array of varied width for linearly polarized light with wavelength of 632.8 nm^19^. The focal line was super-oscillatory with a full-width-at-half-maximum (FWHM) of 240 nm, and the maximum sidelobe was only 10.6% of the central peak, but its focal length was only 40λ. As pointed out in the reference^17^, super-oscillation is a result of precise interference. Therefore, introducing phase into the lens transmittance function is a reasonable and attractive proposition for sub-diffraction photonic devices^17^,^20^. Although phase structures have been applied in conventional micro-lenses design in various ways^21^,^22^,^23^,^24^, the focus size is still within the diffraction limit. A theoretical lens design, based on amplitude and phase modulation, was proposed using optimization-free methods^17^, but its field of view was only 15 wavelengths due to the large sidelobe outside. Although, super-oscillatory focusing lenses and methods have shown great potential in super-resolution microscopy^7^,^8^,^14^ and super-resolution imaging^16^, the reported super-oscillatory lenses have very short focal length (<30λ), large numerical aperture (about 0.9), and very small field of view (<20λ). For far-field super-resolution applications, it is of great interest in developing super-oscillatory devices with long focal length, large field of view and small sidelobes to greatly enhance their performance. To address the problem, here, we reported an ultra-long focal length planar lens with comparative small numerical aperture based on binary phase and amplitude modulation for focusing of circularly polarized plane wave. A focal spot size beyond the diffraction limit and the super-oscillation criterion was achieved without compromising the performance of small sidelobes, leading to super-oscillatory focusing with a large field of view.

## Materials and Methods

**Figure 1:**
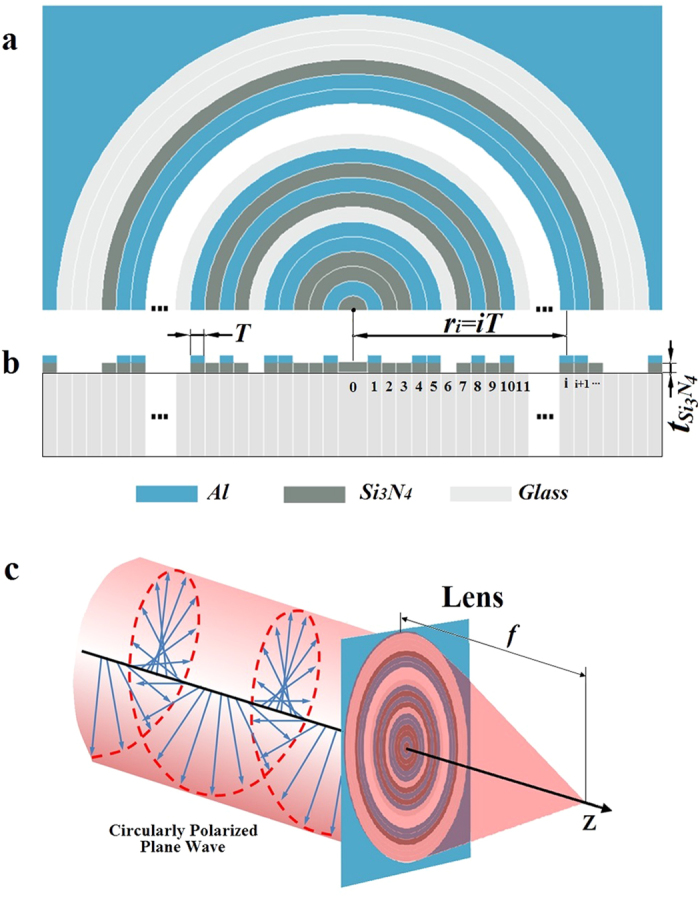
The geometrical structure of the binary amplitude-phase lens showing (a) the top view of the ring structure on the lens and (b) the cross-section of the lens, and (c) the focusing of circularly polarized plane wave.

The structure of the lens is presented in Fig. 1. Figure 1(a) is the top view of the lens, which consists of a series of concentric rings. The width of each ring was a fixed value of *T*. Due to symmetry, only upper half of the lens is illustrated in the figure. As shown in Fig. 1(b), the rings comprise basically two different types of layers, one an aluminum layer and the other a Si_3_N_4_ layer, grown on a glass substrate. The 100-nm aluminum film was grown on the top of Si_3_N_4_ layer; the former was used to realize the binary amplitude modulation of the incident wave and the latter to achieve binary phase modulation. Such structures can generate three different (amplitude, phase) combinations, namely: (0, 0), (1, 0) and (1, *φ*). The thickness of Si_3_N_4_ layer *t*_Si3N4_ was determined by the relative phase delay *φ* and the refractive index of Si_3_N_4_ *n*_Si3N4_, which can be expressed by *t*_Si3N4_ = *φλ*/2π(*n*_Si3N4_ − 1).

Utilizing vectorial angular spectrum method^25^,^26^ and genetic algorithms^27^, a point-focusing lens was designed, based on binary amplitude (0, 1) and binary phase (0, π) mask for circularly polarized plane wave at wavelength λ = 632.8 nm. According to the vectorial angular spectrum method, for circularly polarization wave, the electrical components of the diffraction field on the focal plane at z = z_*f*_ can be calculated by using the following equations (1).


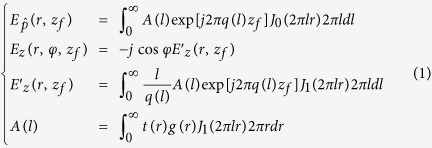


where 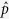 denotes the polarization direction of the incident light, *g*(*r*) and *t*(*r*) are the incident beam profile and transmittance function of the lens, *J*_*0*_ and *J*_*1*_ are the zero- and first-order Bessel functions, *q*(*l*) = (1/λ^2^ − *l*^2^)^1/2^, and *φ* is the angular coordinate with respect to the polarization direction 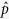. The incident wave with circular polarization had two vectorial electrical components on the focal plane: the 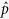 component 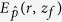 rotating with the electrical vector of the circularly polarized wave, and the longitude component 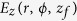 along the optical axis, or the z axis. In the circularly polarized wave, 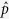 rotates with the incident electrical vector at a constant frequency. Therefore, time average optical intensity distribution on the focal plane can be expressed in 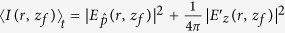. Due to this rotation property of the circularly polarized light, the effective intensity distribution generated by the longitude component is greatly reduced due to the factor of 1/4π, which helps to reduce the sidelobe intensity and generate a circular-symmetrical intensity distribution on the focal plane, as shown later. This is the advantage to use circularly polarized light instead of linear polarized light for focusing.

**Figure 2:**
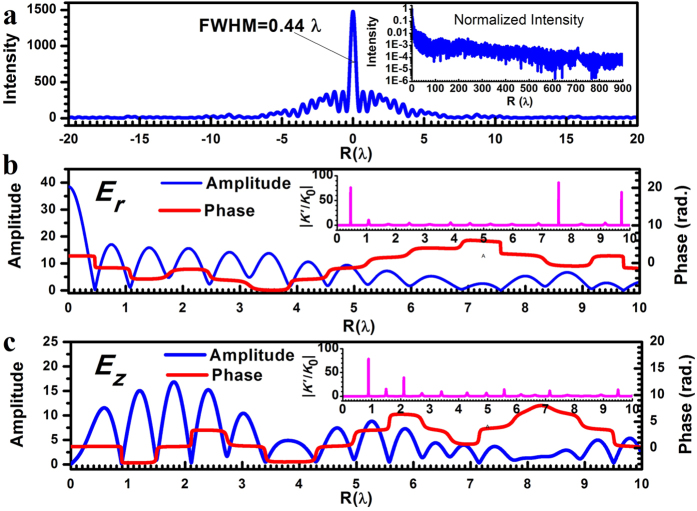
The optimized (a) optical intensity, (b) radial electrical component, and (c) longitude electrical component on the focal plane; inset (a) shows normalized optical intensity in logarithmic scale; insets (b,c) show the local wavenumber distribution for radial and longitude electrical components, respectively.

The radius of the lens is *R*_lens_ = 500λ (316.4 μm), which consists of 633 concentric rings with ring width *T* = 500 nm. The focal length is *f* = 400λ with numerical aperture NA = 0.781, diffraction limit 0.64λ (0.5λ/NA), and super-oscillation criterion 0.487λ (0.38λ/NA). Following the theoretical design, the focal plane optical intensity distribution of the lens is illustrated in Fig. 2(a), which shows a focal spot with a FWHM of 0.44λ, smaller than both the diffraction limit and super-oscillation criterion. The largest sidelobe is located in the nearby region of the focus and its intensity is only 25% of the central lobe. Also, no other large side lobes are noticed in the area of [−900λ, +900λ], as shown in the inset of Fig. 2(a), which gives the normalized optical intensity on the focal plane. At the point 1.5λ away from the center, the intensity drop to 14% of the central lobe intensity, and at the point 8λ away from the center, the intensity is only 3.5% of the central lobe. In the region beyond 16λ, the optical intensity becomes less than 1.7% of the central lobe. Actually, according to numerical calculation, the total transmitted energy was exactly equal to the total energy in the area of [−900λ, +900λ] on the focal plane; therefore, there should be no other sidelobes outside this region.

Figure 2(b,c) show the theoretical amplitude and phase distribution for the electrical field components 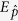 and *E*_*z*_ respectively. It was found that only 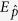 contributed to the focal spot central lobe, while *E*_*z*_ only added sidelobes to the focal plane. Therefore, the time-average of |*E*_*z*_|^2^ only lead to sidelobe suppression without reducing the major lobe intensity. In the phase distribution, the phase changed sharply at several points, and the phase was almost inversed. This led to a large local wavenumber *k′*, as shown in the insets of Fig. 2(b,c), where *k*_*0*_ is the wavenumber in vacuum. At those points, the absolute value of local wavenumber is much larger than *k*_*0*_, and this constituted the direct evidence of super-oscillation. Substituting the electrical field *E* (*x*, *y*, *z*) = *A*(*x, y, z*) exp {*jφ*(*x, y, z*)} in each individual polarization into the Helmholtz wave equation, we obtained following equations for the electrical field amplitude *A*(*x, y, z*) and phase *φ*(*x, y, z*) distribution for each individual polarization respectively.


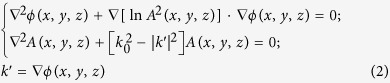


As shown in Fig. 2(b), at the point of the first valley, next to the central lobe at *R* = 0.46λ, the value of local wavenumber *k′* is 73 times larger than *k*_*0*_. According to the second equation in equations (2), the large *k′* value results in a sharp damp in the local electrical amplitude, and therefore shapes the sub-diffraction central lobe.

**Figure 3:**
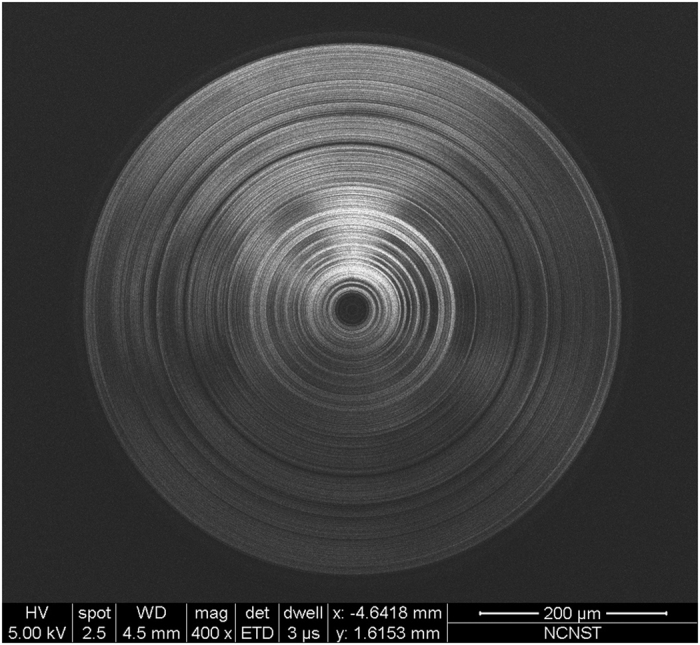
The SEM image of the microlens.

Following the theoretical design, a micro lens was fabricated using electron-beam lithography and dry etching. The detailed geometry of the micro-lens can be found in the Table 1 in the Supplementary Information. The lens substrate was a 500-μm thick Pyrex 7740 glass. A Si_3_N_4_ layer was first deposited on the substrate with PECVD coating. The refractive index of the Si_3_N_4_ layer was characterized with an ellipsometry, which gave a refractive index of 1.91. The thickness of this dielectric layer was about 350 nm to achieve a phase difference of π respect to the phase delay of an optical length, equal to the dielectric layer thickness, in vacuum. A 100-nm thick aluminum film was grown on the top of Si_3_N_4_ layer by sputter coating. Then ring structures were patterned with electron-beam-lithography. Finally, dry etching was employed to create aluminum and Si_3_N_4_ dielectric ring structures respectively. Figure 3 is the SEM image of the microlens.

To experimentally investigate the super-oscillatory focusing lens, the optical intensity distribution was obtained through a tapered optical fiber probe (CFN-100 of Nanonics Imaging Ltd.) mounted on a 3-D piezo nanopositioner (P-561.3CD of Physik Instrumente GmbH & Co.). The probe diameter was 100 nm. The spatial resolution and scanning range of the nanopositioner were about 10 nm and 100 μm for each of the x, y and z axes respectively. The illumination light source was a 10-mW He-Ne laser (632.8 nm) with linear polarization. A quarter-wave plate was put into the laser beam to convert it into circularly polarized wave. The laser beam diameter was expanded to 3 mm to ensure a uniform illumination within the whole lens working area. The lens was mounted on a home-made holder and located at the beam center. The photons collected by the probe were sent into a single photon detector (SPCM50A/M of Thorlabs Inc.). By controlling the 3-D nanopositioner, the fiber probe can be set at different distances from the micro-lens surface along the z-axis, and scanned to obtain the xy-plane optical intensity distribution at different distances. Compared with focusing characterization using microscope imaging system, whose field of view is quite small at high resolution mode, the one based on nano-fiber probe and nanopositioner can obtain the optical intensity with high spatial resolution in a comparative larger area. Besides, microscope imaging system has difficulty in measuring the super-oscillatory focal spot with a very long focal length, because its small field of view might fail to collect optical field in an area large enough to regenerate a correct focal spot image.

## Results and Discussions

**Figure 4:**
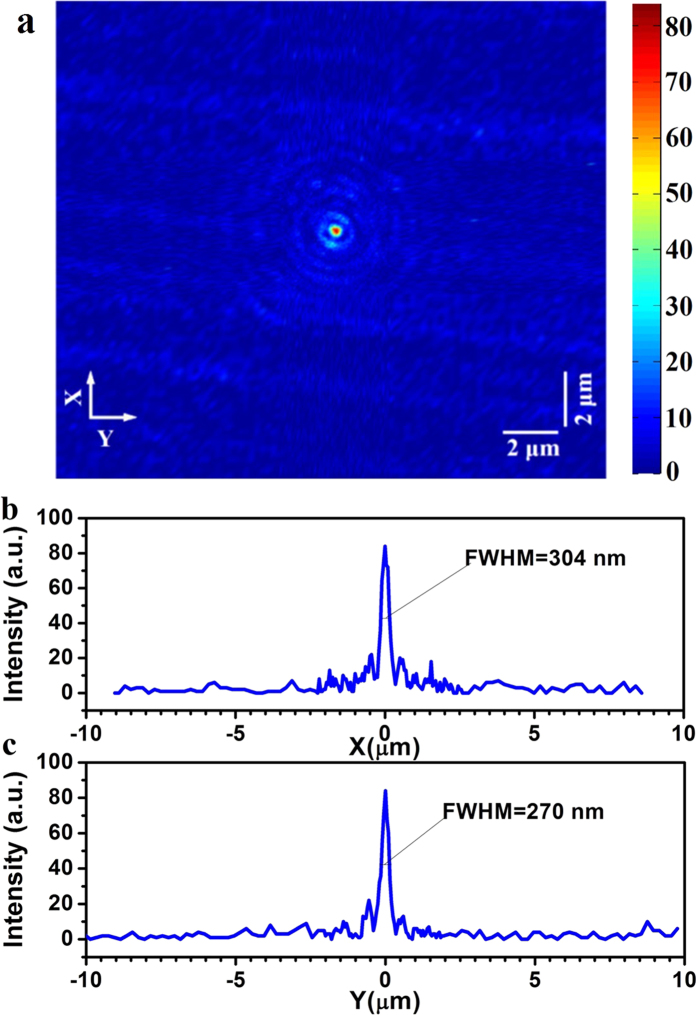
(**a**) The color map of the focal plane intensity distribution; (**b**) the intensity distribution along the x-axis; and **(c)** the intensity distribution along the y-axis.

**Figure 5:**
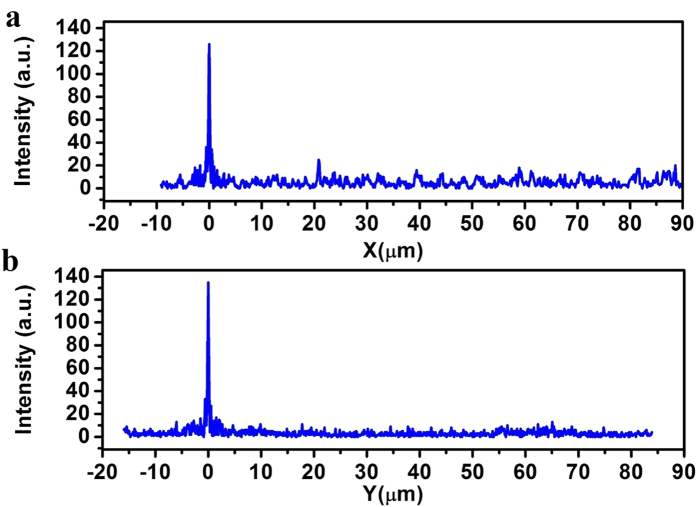
(**a**) the intensity distribution in [−10 μm, +90 μm] along the x-axis; and (**b**) the intensity distribution in [−16 μm, +84 μm] along the y-axis.

In the experiments, a focal spot was found 252.8 μm away from the lens output surface, which is in good agreement with the theoretical focal length 253.12 μm. The focal plane optical intensity was obtained for an area of 20 × 20 μm^2^, with the focal spot located at the center, as depicted in Fig. 4(a). Several weak sidelobe rings were found surrounding the focal spot, but no large side lobes were observed in the rest of the map. To see this clearly, the optical intensity distribution was plotted along x-axis and y-axis in Fig. 4(b,c) respectively. It was found that the FWHM of the intensity distributions along x- and y-axes were a little bit different. The FWHM was 304 nm (0.48λ) along x-axis and 270 nm (0.426λ) along y-axis, and both were smaller than the diffraction limit 0.64λ and super-oscillation criterion 0.486λ. The intensity of the largest sidelobe was about 26% of the central lobe, which is close to 25% in the numerical simulation. The focal spot intensity distribution, though not perfect, was nearly circular and symmetrical. The little deviation from circular symmetry is believed to be caused by the mechanic shift during the probe scanning. To evaluate the focal spot size, the intensity distribution was measured along 8 different directions across the central position of the spot in Fig. 4, where the angles between the y-axis and the directions of intensity distribution measurement were 0, π/8, π/4, 3π/8, π/2, 5π/8, 3π/4, and 7π/8. An FWHM of 287 nm (0.454λ) was obtained by averaging the FWHMs of all the 8 curves, which is similar with the FWHM of 278 nm in the design. The FWHM was just between the two values of 304 nm and 270 nm obtained along the x-axis and y-axis in Fig. 4(b,c). According to the super-oscillation criteria 0.486λ, the focal spot is already in the super-oscillatory dormain. To understand the sidelobe distribution over a larger area, in the allowed scan range, two 100-μm scans were conducted along x-axis and y-axis respectively, as depicted in Fig. 5(a,b). In the measured areas, there were no other large sidelobes, which agreed with the foregoing theoretical simulation, shown in the inset of Fig. 2(a). We also noticed that the intensity was a little bit larger than the theoretical prediction in the area with radius larger than 10λ, which was majorly caused by the noise in the experiment. Therefore, this planar lens based on binary amplitude-phase modulation has an ultra-long focal length of 252.8 μm and comparative small numerical aperture of 0.78, which can achieve super-oscillatory focusing with FWHM of 0.454λ and small sidelobs. At least in the area within the radius of 90 μm (142λ), the largest side lobe was less than 26% of the central peak. The experiment results are similar with the theoretical design, which indicates that it is possible to have a lens with ultra-long focal length, and super-oscillatory focusing performance, but small sidelobes in a very large area.

**Figure 6:**
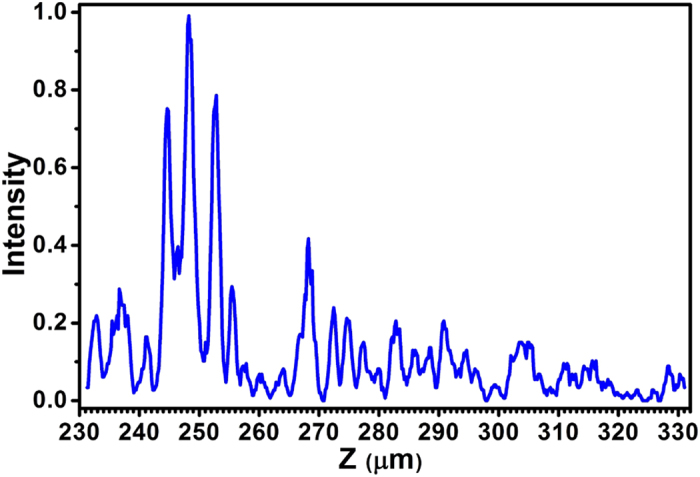
The optical intensity along the optical axis.

To investigate the optical intensity distribution on the optical axis, a 100 μm-scan was performed along the z-axis. As plotted in Fig. 6, three major peaks were found on the optical axis. Besides the expected focal spot at 252.8 μm, two other strong focal spots were located at 244.6 μm and 248.2 μm. These two spots were somewhat jointed, while the theoretically predicted focal spot showed no connection with the other two. The spot at 248.2 μm had stronger intensity than the other two spots and its average FWHM was about 340 nm, which is not super-oscillatory, but smaller than the diffraction limit 405 nm (0.64λ). It was also interesting to notice that many small peaks were distributed in the whole 100 μm range along the z-axis.

The light throughput is one of the key parameter for a focusing lens. As pointed out in the reference^17^, a phase mask will lead to a better optical efficiency compared with pure metallic amplitude mask. Our lens based on binary amplitude and phase, which utilizes transparent dielectric materials for phase modulation, is expected to enhance the optical efficiency of focusing. Although the lens has ultra-long focal length of 252.8 μm and comparative small numerical aperture of 0.78, it realized a super-oscillatory focusing with focal spot FWHM of 0.454λ, which can be further improved by introducing more phase values between 0 and 2π in the lens design and fabrication.

## Conclusions

In summary, sub-diffraction photonic device suffers from huge sidelobes around its focal spot. It’s a challenge to realized super-oscillatory focusing while suppressing sidelobes in a large area, particularly in the case where focal spot is less than the super-oscillation criteria 0.38*λ*/NA. To address the problem, an ultra-long focal length super-oscillatory lens based on binary amplitude and binary phase modulation was proposed and fabricated for circularly polarized light at wavelength of 632.8 nm. The radius of the micro lens was 500λ, the focal length was 400λ, and the numerical aperture was 0.781. Using 100-nm taped fiber probe, 3D nanopositioner and single photon detector, the optical intensity distribution on the lens focal plane was measured with illuminating by circularly polarized plane wave. Experimental results showed that the focal length was about 252.8 μm and the focal spot full width at half maximum was 287nm (0.454λ), which is smaller than the diffraction limit 0.64λ (0.5λ/NA) and the super-oscillation criterion 0.487λ (0.38λ/NA). Moreover, on the focal plane, within the radius of 142λ, the ratio between largest sidelobe and the central lobe is only 26%, leaving a wide field of view. Therefore, we theoretically and experimentally demonstrated the possibility of realizing super-oscillatory focusing with small sidelobes by a planar lens with ultra-long focal length and small numerical aperture. The focal spot can be further reduced with a larger numerical aperture by either increasing the lens radius or decreasing the focal length. The sidelobes are also expected to be further suppressed by applying more phase values between 0 and 2π in the lens design and fabrication^20^. Such super-oscillatory focusing with ultra-long focal length, small sidelobes and wide field of view is attractive for applications in super-resolution optical microscopy^7^,^8^,^14^, super-resolution optical imaging^16^, stimulated emission depletion microscopy^28^, ultra-high-density optical storage, nano-fabrication^29^ and so on.

## Additional Information

**How to cite this article**: Chen, G. *et al*. Super-oscillatory focusing of circularly polarized light by ultra-long focal length planar lens based on binary amplitude-phase modulation. *Sci. Rep*. **6**, 29068; doi: 10.1038/srep29068 (2016).

## Supplementary Material

Supplementary Information
