# Supplementary material for: Super-oscillatory focusing of circularly polarized light by ultra-long focal length planar lens based on binary amplitude-phase modulation
Source: Sci Rep. 2016 Jun 29;6:29068. doi: 10.1038/srep29068 (PMC4926254; doi:10.1038/srep29068)
Supplement: Supplementary Information [file srep29068-s1.pdf]

Super-oscillatory Focusing of Circularly Polarized Light by Ultra-long Focal Length Planar Lens based on Binary Amplitude-phase Modulation

Gang Chen[1], Yuyan Li[1], Anping Yu[1], Zhongquan Wen[1], Luru Dai [2], Li Chen[1], Zhihai Zhang[1], Senlin Jiang[1], Kun Zhang[1,2], Xianyou Wang[2], Feng Lin [3]

[1]Key Laboratory of Optoelectronic Technology and Systems (Chongqing University), Ministry of Education, and Key Disciplines Lab of Novel Micro-nano Devices and System Technology, Chongqing University,174 Shazheng Street, Shapingba, Chongqing 400044, China, [2]National Center for Nanoscience and Technology, No.11 Zhong Guan CunBei Yi Tiao, Beijing 100190, China, [3]School of Physics, State Key Laboratory for Mesoscopic Physics, Peking University, Beijing 100871, China

Supplementary

Table 1. the geometry of the micro-lens (T=500 nm)

i	Ri (nm)	t_{Si3N4} (nm)	t_{Al} (nm)	i	Ri (nm)	t_{Si3N4} (nm)	t_{Al} (nm)	i	Ri (nm)	t_{Si3N4} (nm)	t_{Al} (nm)
0	0	348	0	48	24000	348	100	96	48000	348	100
1	500	348	100	49	24500	0	0	97	48500	0	0
2	1000	348	0	50	25000	348	100	98	49000	0	0
3	1500	348	0	51	25500	0	0	99	49500	0	0
4	2000	348	100	52	26000	348	0	100	50000	348	100
5	2500	348	100	53	26500	0	0	101	50500	348	0
6	3000	0	0	54	27000	348	100	102	51000	348	100
7	3500	348	0	55	27500	348	100	103	51500	348	100
8	4000	348	100	56	28000	348	100	104	52000	348	100
9	4500	348	0	57	28500	348	100	105	52500	348	0
10	5000	348	100	58	29000	348	100	106	53000	348	100
11	5500	0	0	59	29500	348	100	107	53500	0	0
12	6000	0	0	60	30000	348	100	108	54000	0	0
13	6500	348	100	61	30500	348	100	109	54500	348	0
14	7000	0	0	62	31000	0	0	110	55000	348	0
15	7500	348	100	63	31500	348	0	111	55500	348	0
16	8000	348	100	64	32000	348	100	112	56000	0	0
17	8500	348	100	65	32500	0	0	113	56500	348	0
18	9000	348	100	66	33000	348	0	114	57000	348	100
19	9500	348	0	67	33500	348	100	115	57500	348	0
20	10000	348	0	68	34000	348	0	116	58000	348	100
21	10500	348	100	69	34500	348	100	117	58500	348	100
22	11000	348	100	70	35000	348	0	118	59000	348	100
23	11500	348	0	71	35500	348	100	119	59500	348	100
24	12000	348	100	72	36000	348	100	120	60000	348	100
25	12500	348	100	73	36500	348	73	121	60500	348	100
26	13000	348	0	74	37000	348	0	122	61000	348	100
27	13500	348	100	75	37500	348	100	123	61500	0	0
28	14000	348	100	76	38000	348	100	124	62000	348	0
29	14500	0	0	77	38500	0	0	125	62500	0	0
30	15000	348	100	78	39000	348	100	126	63000	348	0
31	15500	348	0	79	39500	0	0	127	63500	0	0
32	16000	348	0	80	40000	348	100	128	64000	348	0
33	16500	348	0	81	40500	348	100	129	64500	0	0
34	17000	348	0	82	41000	348	0	130	65000	348	100
35	17500	0	0	83	41500	348	100	131	65500	0	0
36	18000	348	0	84	42000	348	100	132	66000	348	100
37	18500	348	0	85	42500	0	0	133	66500	348	0
38	19000	348	0	86	43000	348	100	134	67000	348	0
39	19500	348	100	87	43500	0	0	135	67500	348	0
40	20000	348	100	88	44000	0	0	136	68000	348	100
41	20500	348	0	89	44500	0	0	137	68500	0	0
42	21000	348	100	90	45000	348	0	138	69000	348	100
43	21500	348	100	91	45500	0	0	139	69500	348	0
44	22000	348	100	92	46000	348	100	140	70000	0	0
45	22500	348	100	93	46500	348	100	141	70500	348	100
46	23000	348	0	94	47000	0	0	142	71000	348	0
47	23500	0	0	95	47500	348	100	143	71500	348	100

i	Ri (nm)	t_{Si3N4} (nm)	t_{Al} (nm)	i	Ri (nm)	t_{Si3N4} (nm)	t_{Al} (nm)	i	Ri (nm)	t_{Si3N4} (nm)	t_{Al} (nm)
144	72000	348	0	192	96000	348	100	240	120000	0	0
145	72500	348	100	193	96500	0	0	241	120500	0	0
146	73000	0	0	194	97000	348	100	242	121000	348	0
147	73500	348	100	195	97500	348	100	243	121500	348	100
148	74000	348	100	196	98000	348	0	244	122000	348	100
149	74500	348	100	197	98500	0	0	245	122500	0	0
150	75000	348	0	198	99000	348	100	246	123000	348	100
151	75500	348	0	199	99500	348	100	247	123500	348	0
152	76000	348	0	200	100000	348	100	248	124000	348	0
153	76500	348	100	201	100500	348	100	249	124500	0	0
154	77000	348	0	202	101000	348	100	250	125000	0	0
155	77500	0	0	203	101500	348	100	251	125500	348	0
156	78000	348	100	204	102000	348	100	252	126000	348	0
157	78500	348	100	205	102500	348	100	253	126500	0	0
158	79000	348	100	206	103000	348	0	254	127000	348	100
159	79500	348	0	207	103500	348	100	255	127500	348	0
160	80000	0	0	208	104000	348	100	256	128000	348	0
161	80500	348	100	209	104500	0	0	257	128500	348	100
162	81000	0	0	210	105000	0	0	258	129000	348	100
163	81500	348	100	211	105500	348	100	259	129500	0	0
164	82000	0	0	212	106000	348	100	260	130000	348	100
165	82500	0	0	213	106500	348	0	261	130500	348	0
166	83000	0	0	214	107000	348	100	262	131000	348	0
167	83500	348	100	215	107500	348	100	263	131500	348	100
168	84000	348	100	216	108000	348	100	264	132000	0	0
169	84500	348	100	217	108500	348	100	265	132500	348	0
170	85000	348	100	218	109000	0	0	266	133000	0	0
171	85500	348	0	219	109500	348	0	267	133500	348	100
172	86000	348	100	220	110000	348	100	268	134000	0	0
173	86500	348	0	221	110500	0	0	269	134500	348	100
174	87000	348	0	222	111000	0	0	270	135000	0	0
175	87500	348	100	223	111500	348	100	271	135500	348	100
176	88000	348	0	224	112000	348	100	272	136000	0	0
177	88500	348	100	225	112500	348	100	273	136500	348	100
178	89000	348	100	226	113000	348	0	274	137000	348	100
179	89500	0	0	227	113500	348	100	275	137500	348	0
180	90000	348	0	228	114000	348	0	276	138000	348	0
181	90500	348	0	229	114500	348	0	277	138500	0	0
182	91000	348	100	230	115000	348	100	278	139000	348	100
183	91500	0	0	231	115500	348	0	279	139500	348	100
184	92000	0	0	232	116000	348	100	280	140000	348	100
185	92500	348	0	233	116500	348	100	281	140500	348	100
186	93000	0	0	234	117000	348	0	282	141000	0	0
187	93500	348	0	235	117500	348	100	283	141500	348	0
188	94000	0	0	236	118000	0	0	284	142000	0	0
189	94500	348	100	237	118500	348	100	285	142500	348	0
190	95000	348	100	238	119000	348	100	286	143000	348	0
191	95500	348	100	239	119500	348	0	287	143500	348	100

i	Ri (nm)	t_{Si3N4} (nm)	t_{Al} (nm)	i	Ri (nm)	t_{Si3N4} (nm)	t_{Al} (nm)	i	Ri (nm)	t_{Si3N4} (nm)	t_{Al} (nm)
288	144000	348	0	337	168500	348	100	386	193000	0	0
289	144500	348	100	338	169000	348	100	387	193500	348	100
290	145000	348	100	339	169500	0	0	388	194000	0	0
291	145500	348	0	340	170000	0	0	389	194500	348	0
292	146000	348	0	341	170500	0	0	390	195000	348	100
293	146500	348	100	342	171000	348	100	391	195500	348	0
294	147000	0	0	343	171500	348	100	392	196000	0	0
295	147500	348	100	344	172000	348	100	393	196500	348	0
296	148000	348	100	345	172500	348	100	394	197000	348	100
297	148500	0	0	346	173000	348	0	395	197500	348	100
298	149000	348	100	347	173500	348	0	396	198000	0	0
299	149500	348	100	348	174000	348	100	397	198500	0	0
300	150000	348	100	349	174500	0	0	398	199000	348	100
301	150500	348	100	350	175000	0	0	399	199500	348	0
302	151000	348	100	351	175500	348	100	400	200000	348	0
303	151500	0	0	352	176000	348	100	401	200500	348	0
304	152000	348	100	353	176500	348	0	402	201000	348	100
305	152500	348	0	354	177000	348	100	403	201500	0	0
306	153000	348	100	355	177500	348	0	404	202000	0	0
307	153500	0	0	356	178000	348	100	405	202500	348	100
308	154000	0	0	357	178500	348	100	406	203000	348	100
309	154500	348	0	358	179000	0	0	407	203500	348	100
310	155000	348	0	359	179500	348	100	408	204000	0	0
311	155500	348	100	360	180000	348	0	409	204500	0	0
312	156000	0	0	361	180500	348	100	410	205000	348	100
313	156500	348	100	362	181000	348	0	411	205500	348	100
314	157000	348	100	363	181500	0	0	412	206000	348	0
315	157500	348	0	364	182000	348	0	413	206500	348	100
316	158000	0	0	365	182500	348	0	414	207000	348	0
317	158500	348	100	366	183000	348	0	415	207500	348	0
318	159000	348	0	367	183500	348	0	416	208000	348	0
319	159500	348	0	368	184000	348	100	417	208500	0	0
320	160000	348	100	369	184500	348	100	418	209000	0	0
321	160500	0	0	370	185000	348	100	419	209500	348	0
322	161000	348	100	371	185500	0	0	420	210000	348	0
323	161500	348	0	372	186000	348	100	421	210500	0	0
324	162000	348	0	373	186500	0	0	422	211000	0	0
325	162500	348	0	374	187000	0	0	423	211500	0	0
326	163000	348	100	375	187500	348	0	424	212000	348	100
327	163500	0	0	376	188000	348	0	425	212500	348	0
328	164000	348	0	377	188500	348	100	426	213000	0	0
329	164500	348	0	378	189000	0	0	427	213500	0	0
330	165000	348	100	379	189500	348	0	428	214000	348	0
331	165500	348	100	380	190000	0	0	429	214500	348	0
332	166000	348	100	381	190500	348	0	430	215000	0	0
333	166500	348	100	382	191000	0	0	431	215500	0	0
334	167000	348	0	383	191500	348	100	432	216000	348	100
335	167500	0	0	384	192000	348	0	433	216500	348	0
336	168000	348	100	385	192500	348	0	434	217000	348	100

i	Ri (nm)	t_{Si3N4} (nm)	t_{Al} (nm)	i	Ri (nm)	t_{Si3N4} (nm)	t_{Al} (nm)	i	Ri (nm)	t_{Si3N4} (nm)	t_{Al} (nm)
435	217500	0	0	484	242000	348	100	533	266500	348	0
436	218000	0	0	485	242500	348	100	534	267000	348	100
437	218500	348	100	486	243000	348	0	535	267500	348	0
438	219000	348	100	487	243500	348	100	536	268000	0	0
439	219500	348	0	488	244000	348	100	537	268500	348	100
440	220000	348	100	489	244500	348	100	538	269000	348	100
441	220500	0	0	490	245000	348	100	539	269500	348	0
442	221000	348	100	491	245500	348	0	540	270000	348	100
443	221500	348	0	492	246000	348	100	541	270500	0	0
444	222000	348	100	493	246500	0	0	542	271000	348	0
445	222500	0	0	494	247000	348	100	543	271500	348	100
446	223000	0	0	495	247500	348	0	544	272000	348	0
447	223500	348	100	496	248000	348	100	545	272500	0	0
448	224000	348	100	497	248500	348	100	546	273000	0	0
449	224500	0	0	498	249000	348	0	547	273500	348	0
450	225000	0	0	499	249500	0	0	548	274000	348	100
451	225500	348	100	500	250000	348	0	549	274500	348	0
452	226000	348	0	501	250500	348	0	550	275000	348	100
453	226500	348	100	502	251000	0	0	551	275500	348	100
454	227000	0	0	503	251500	348	0	552	276000	348	0
455	227500	0	0	504	252000	0	0	553	276500	348	100
456	228000	348	0	505	252500	348	100	554	277000	0	0
457	228500	348	0	506	253000	348	100	555	277500	0	0
458	229000	348	100	507	253500	348	0	556	278000	348	100
459	229500	0	0	508	254000	0	0	557	278500	348	0
460	230000	0	0	509	254500	348	0	558	279000	348	100
461	230500	348	0	510	255000	348	0	559	279500	348	100
462	231000	348	100	511	255500	0	0	560	280000	0	0
463	231500	348	100	512	256000	0	0	561	280500	348	0
464	232000	0	0	513	256500	348	100	562	281000	348	0
465	232500	0	0	514	257000	348	100	563	281500	0	0
466	233000	348	100	515	257500	0	0	564	282000	348	0
467	233500	348	0	516	258000	0	0	565	282500	348	100
468	234000	348	100	517	258500	348	100	566	283000	348	0
469	234500	0	0	518	259000	348	100	567	283500	348	100
470	235000	348	100	519	259500	348	100	568	284000	348	100
471	235500	348	0	520	260000	348	0	569	284500	0	0
472	236000	348	100	521	260500	348	100	570	285000	0	0
473	236500	0	0	522	261000	0	0	571	285500	348	0
474	237000	0	0	523	261500	348	100	572	286000	348	100
475	237500	348	100	524	262000	348	0	573	286500	348	100
476	238000	348	0	525	262500	348	100	574	287000	0	0
477	238500	348	0	526	263000	0	0	575	287500	348	100
478	239000	0	0	527	263500	348	100	576	288000	348	0
479	239500	0	0	528	264000	348	0	577	288500	0	0
480	240000	348	100	529	264500	348	100	578	289000	348	0
481	240500	348	0	530	265000	348	0	579	289500	348	100
482	241000	348	100	531	265500	0	0	580	290000	348	0
483	241500	0	0	532	266000	0	0	581	290500	348	0

i	Ri (nm)	t_{Si3N4} (nm)	t_{Al} (nm)	i	Ri (nm)	t_{Si3N4} (nm)	t_{Al} (nm)	i	Ri (nm)	t_{Si3N4} (nm)	t_{Al} (nm)
582	291000	0	0	599	299500	0	0	616	308000	348	0
583	291500	348	0	600	300000	348	100	617	308500	348	100
584	292000	0	0	601	300500	348	100	618	309000	348	0
585	292500	348	0	602	301000	0	0	619	309500	348	0
586	293000	348	0	603	301500	348	100	620	310000	0	0
587	293500	0	0	604	302000	348	100	621	310500	348	0
588	294000	348	100	605	302500	348	0	622	311000	0	0
589	294500	0	0	606	303000	348	0	623	311500	348	0
590	295000	348	100	607	303500	0	0	624	312000	348	0
591	295500	348	0	608	304000	348	0	625	312500	0	0
592	296000	348	100	609	304500	0	0	626	313000	348	100
593	296500	348	0	610	305000	348	100	627	313500	348	100
594	297000	0	0	611	305500	348	0	628	314000	348	100
595	297500	348	0	612	306000	0	0	629	314500	348	0
596	298000	348	100	613	306500	348	100	630	315000	0	0
597	298500	348	100	614	307000	348	100	631	315500	0	0
598	299000	348	0	615	307500	348	100	632	316000	0	0
